# Quantification of riverine macroplastics in a farmland area in Japan

**DOI:** 10.1007/s11356-025-36160-6

**Published:** 2025-03-01

**Authors:** Zahura Chowdhury, Kuriko Yokota, Nguyen Minh Ngoc, Takanobu Inoue

**Affiliations:** Department of Architecture and Civil Engineering, Toyohashi University of Technology, 1-1 Hibarigaoka, Tempaku, Toyohashi, Aichi 441-8580 Japan

**Keywords:** Macroplastics, Sunny, Rainy, Agricultural land use, Polyethylene, Mulch

## Abstract

Plastic pollution has become a major environmental threat, with extensive research focusing on the transfer of plastic waste from urban areas to oceans. However, limited attention has been given to the role of agricultural areas in plastic emissions to rivers. Rivers play a crucial role in transporting plastics to the ocean. This study investigates plastic waste pollution in the Hamada River, which drains into Mikawa Bay, with a particular focus on how agricultural activities and weather conditions affect plastic distribution. We collected macroplastics from both the river and its banks under sunny and rainy conditions. On sunny days, lightweight plastics, especially film-shaped items, dominated the river’s waste. In contrast, rainy weather led to greater accumulation of plastics along the riverbank, with a broader diversity of shapes and colors. Plastic mulch was the predominant type of waste found in both the river and along the banks, with polyethylene as the most common polymer, followed by polypropylene. Notably, no PET plastics were detected in the river, contrasting with marine environments. These findings suggest that weather patterns and the proximity of agricultural sources significantly influence plastic distribution in rivers. Future research should explore the long-term dynamics of plastic transport, especially during high-flow events, to better understand the impact of plastic pollution on river ecosystems and the wider environment.

**Supplementary Information:**

The online version contains supplementary material available at 10.1007/s11356-025-36160-6.

## Introduction

Plastic pollution has become a pressing global environmental issue, with an estimated 5.25 trillion pieces of plastic debris, weighing over 250,000 t, found in the ocean (Eriksen et al. 2014; Parker 2023). The global production of plastics reached approximately 460 million tons in 2019, and this number is expected to triple by 2060 (Kottasová 2023; Ritchie et al. 2023). Most of this plastic waste ends up in the environment with single-use plastics contributing significantly to the growing problem (Barnes et al. 2009). Despite efforts to reduce waste, Japan ranks second in the world for per capita plastic waste emissions (IGES –, 2022), highlighting the urgent need for improved waste management strategies.

Rivers play a crucial role in transporting plastic waste from land to the oceans, but the impact of plastic pollution on riverine ecosystems is also significant. Macroplastics, plastics larger than 5 cm, accumulate in rivers over time, clogging waterways and increasing the risk of flooding (van Emmerik et al. 2018; van Emmerik and Schwarz 2020; Jia et al. 2023). The transportation of plastics to rivers is largely driven by urban, industrial and agricultural runoff, particularly during rain events, with the rate of plastic transport increasing significantly during peak discharge or storm flow events (van Emmerik et al. 2019, 2022, 2023). However, the quantity and characteristics of plastic waste vary according to anthropogenic activities, especially in urban areas. For example, street markets, restaurants, and recreational areas often generate large amounts of plastic waste, much of which is improperly disposed of, contributing significantly to plastic pollution in nearby rivers (Franz and Freitas 2012; Kiessling et al. 2019, 2021; Tasseron et al. 2023). While research has explored plastic pollution in urban environments and coastal regions, there is still a limited understanding of plastic waste emissions from agricultural areas, particularly under varying weather conditions. This gap is particularly significant in Japan, where household and industrial plastic waste are well-managed, but plastic debris from agricultural practices is often inadequately collected, resulting in emissions into rivers and surrounding environments (Piehl et al. 2018; Walther et al. 2020).

Given the growing impact of plastic waste on riverine ecosystems, quantifying the emissions of macroplastic waste from farmlands, especially under different weather conditions, is essential for understanding and managing plastic pollution in rivers. This study focuses on quantifying macroplastic waste in riverine ecosystems, particularly from agricultural sources, under both sunny and rainy conditions. These weather conditions are important because they influence how plastic waste is transported and deposited. For instance, during rainy conditions, runoff from surrounding areas can carry plastic debris into rivers and deposit it along the riverbanks, while in sunny conditions, plastics may degrade more quickly and become more mobile in the water column. By studying plastic waste in these different river compartments, collected in rivers during sunny conditions and along riverbanks during rainy conditions, this study aims to assess the role of weather in plastic waste dynamics and identify the factors that contribute to its transport and accumulation in rivers. Plastic debris transported from farmlands to rivers is influenced by various factors, including the plastic’s mass, shape, size, color, item type, and polymer composition. Common polymers found in farmland plastic items include polyethylene (PE), polypropylene (PP), polyethylene terephthalate (PET), polyurethane (PU), polystyrene (PS), and dioctyl phthalate (DOP), each exhibiting different behaviors when exposed to heat and sunlight (Kazemi et al. 2021). These plastics are commonly used in mulching films, packaging items, and foams, with packaging materials (e.g., trays, bags, wraps, bottles, containers) being the most commonly used. With their leakage from these farmlands, some light fragments float on the surface of rivers, while others are heavier and are deposited along the riverbanks. The item characteristics such as shape and color are also indicated to influence the transport behavior of these items, angular shapes for instance with large surface areas may float more easily, and blue plastics often degrade faster under sunlight (Zhao et al. 2022).

To better understand the plastic waste emissions from farmlands to rivers in Japan, we quantified macroplastics based on their physical properties, item types, and polymer compositions under sunny and rainy conditions. We collected plastic waste from both the river and riverbank, paying attention to the role of river cross-sectional profiles, riparian vegetation, and riverbank slopes in determining the deposition or flotation of plastic debris (Bruge et al. 2018; van Emmerik et al. 2018). This study aims to provide valuable data to help anticipate and manage the inputs of plastic waste into rivers, particularly in agricultural areas, under both sunny and rainy conditions. The study was conducted over a time frame from May to November 2022, capturing a range of both sunny and rainy days.

## Methodology

### Study area

**Fig. 1:**
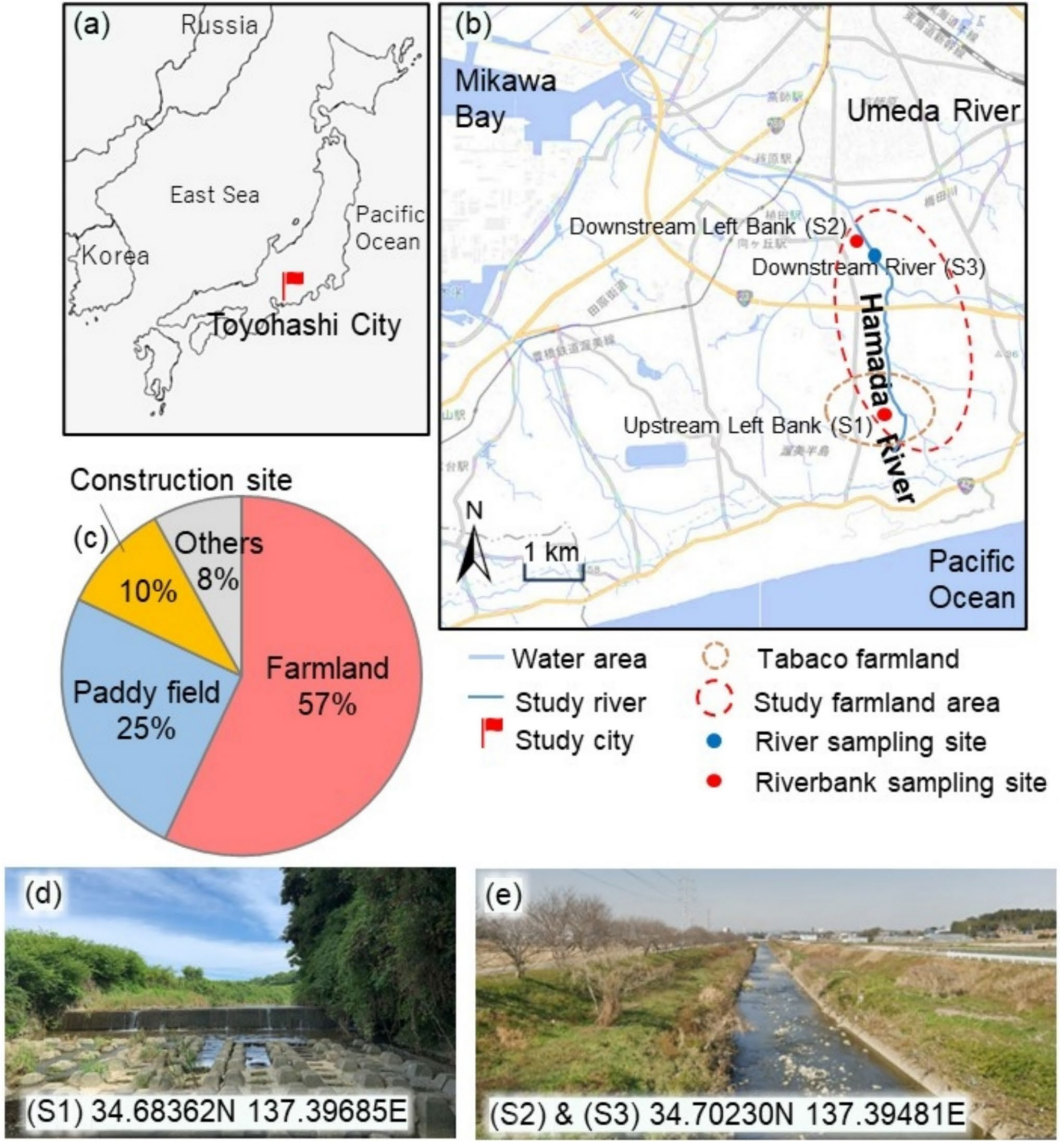
Maps and land use of the sampling area. **a** Location of Japan on the world map. **b** Hamada River and study area within Toyohashi City. **c** Land use distribution in the Hamada River basin. Photos of the sampling sites: **d** upstream left bank (S1); **e** downstream left bank (S2) and downstream river (S3)

The Hamada River (Fig. 1), a tributary of the Umeda River, flows into Mikawa Bay through Toyohashi City, located on the eastern coast of central Japan (Fig. 1a). Toyohashi is a significant agricultural hub in Japan (Toyohashi 2023), with a variety of farms concentrated in the southern fringe of the city’s built-up areas (Ito 1993). The study area along the Hamada River (Fig. 1b), was selected due to its high potential for runoff and plastic waste pollution reaching Mikawa Bay and the Pacific Ocean. Farmlands account for 57% of land use in this area (Fig. 1c), including a tobacco farm located downstream along the river.

The region experiences hot summers and cold, windy winters (Weather Spark 2024), with temperatures typically ranging from 3 to 31 °C. While snow is occasional in the coldest winters, rain is the most common form of precipitation. The rainy season extends from the end of March to mid-October, peaking in early to mid-July with a 50% chance of rainfall.

The region experiences hot summers and cold, windy winters (Weather Spark 2024), with temperatures typically ranging from 3 to 31 °C. While snow is occasional in the coldest winters, rain is the most common form of precipitation. The rainy season lasts from the end of March to mid-October, peaking in early to the mid-July with a 50% probability of rainfall.

In terms of pollution, the Hamada River has been identified as potentially vulnerable to plastic waste pollution, primarily due to runoff from agricultural activities in the surrounding area. Local reports indicate that plastic waste, especially from farming practices, is a significant contributor to pollution in the river (Toyohashi 2022). However, comprehensive pollution statistics specific to plastic debris in the river remain limited, highlighting a gap in monitoring and assessment efforts.

### Macroplastic waste collection

**Fig. 2:**
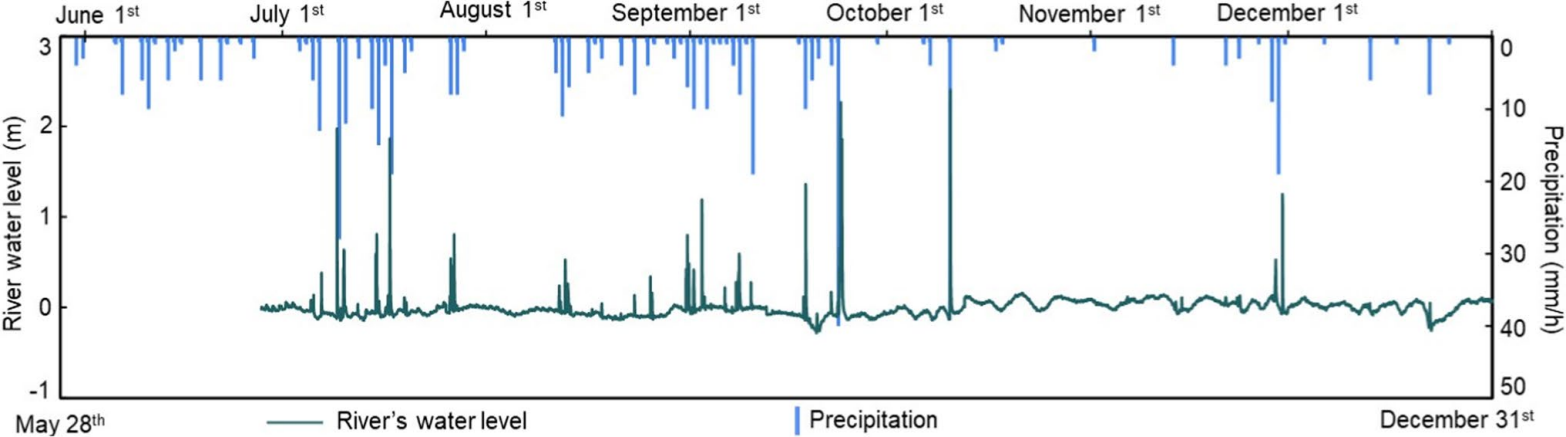
River water levels and precipitation during the study period

In 2002, our team collected deposited plastic waste along the riverbank during rainy conditions and floating plastic waste from the Hamada River during sunny conditions (Fig. 1b, d, e). The total sampling period for this study spanned from the end of May to the end of November, capturing both sunny and rainy days. The sampling following heavy rain events occurred on June 8, July 13, and October 5, during the rainy season in Japan. Rainfall typically begins at the end of May (Fig. 2) and peaks in July (JMA 2022). After these rain events, all deposited plastics and plastic-like objects were collected from two quadrats: a 10.0 m (length) × 0.5 m (width) area on the upstream left bank (S1) and a 10.0 m (length) × 2.5 m (width) area on the downstream left bank (S2). The upstream quadrat was limited in width due to steep hillsides covered in bamboo groves, which lacked accessible entry points (Fig. 1d). Therefore, the sampling site was selected at a point where our team could safely and easily access the riverbank. The downstream sampling locations were chosen near a tobacco farm that extends along the river, to assess the potential impact of agricultural activities on plastic waste emissions.

On October 19, 31, and November 28, floating plastic debris was collected during sunny conditions (Fig. 2). Sampling was conducted for approximately six hours each day using a green nylon net with a mesh size of 3.5 cm × 3.5 cm, suspended across the downstream river (S3) (Fig. 1b, e). The sampling frequency was designed to capture the effects of both sunny conditions and rainfall events on the river’s water level (Fig. 2), allowing for an assessment of how weather conditions influence the transportation and deposition of plastic waste in the river.

All collected samples were carefully sorted from organic matter (such as leaves, grasses, and algae) by hand. The plastic debris were then numbered and transported to the laboratory for washing. After washing, the samples were air-dried and stored at room temperature for further analysis.

### River cross-sectional characteristics

Along with the collection of floating waste, the river’s width was determined by recording the length of the net using a measuring tape. The riverbed was divided into 2-m intervals across the width of the river (Supplementary Figure), and the depth of each segment was measured using a steel ruler. The areas of these segments were then summed to calculate the total cross-sectional area. Flow velocities were measured at the center of each segment, approximately two-thirds of the depth from the riverbed, using a portable electromagnetic flowmeter (LP30, KENEK Co., Ltd., Tokyo, Japan). For each segment, the velocity was recorded as the average of three measurements. The river discharge were estimated by multiplying the cross-sectional area by the mean flow velocity, as shown in the following equation (James and James 2017, USGS 2018).

$$Q=A\times v$$

where *Q* is the river discharge (m^3^/s), *A* is the cross-sectional area (m^2^), and *v* is the mean flow velocity (m/s).

The velocity, depth, and slope of the river were measured to understand their potential effects on the transportation and deposition of plastic waste. Flow velocity, in particular, can influence how far and quickly plastics are transported downstream, with faster flows likely carrying plastics further, while slower velocities may result in their accumulation. The depth of the river affects the distribution of plastics in the water column, as deeper areas may keep plastics floating, whereas shallower areas might lead to deposition. Additionally, the slope of the river impacts the overall flow dynamics, with steeper slopes increasing water velocity and promoting further transportation of plastic debris.

During the field survey, dead water areas with riparian vegetation were also observed. During the field survey, dead water areas with riparian vegetation were also observed. These areas, where water flow is slower or stagnates, may act as sites for plastic accumulation, especially in areas with dense riparian vegetation. Vegetation along the riverbank, such as grasses and shrubs, can physically trap plastics, preventing them from being carried further downstream. Water level data were collected using water loggers (HOBO U20 Water logger, U20-001–04, Onset, MA, USA), providing information on fluctuations in river height over time. Riverbank slope data were obtained through a literature review (Shizuoka 2014, Aichi 2019, Shizuoka 2023) which was used for further understanding of how slope influences plastic movement and deposition along the river.

### Plastic characteristics

The collected plastic waste was visually inspected for shape, size, and aspect ratio. The aspect ratio of each piece was determined by calculating the ratio of length to width. The color was identified by the naked eye. The polymer composition of each sample was identified using Fourier transform infrared spectroscopy (FTIR) with a portable Fourier transform infrared spectrophotometer (Cary 630 FTIR, Agilent, Tokyo, Japan), conducted at a temperature of less than 26 °C and humidity below 50%. The item type was inferred from its color and polymer composition. Each piece was weighed to determine its mass, sorted by mass, and then counted.

### Statistical analysis

To assess variations in macroplastics abundance between the upstream and downstream regions, as well as between the river and riverbank regions, a one-way ANOVA with Tukey’s post hoc test was performed, with a significance level set at a *p* value < 0.05. A *t* test was applied to compare the difference in the percentages of different polymer types.

## Results and discussion

### River cross-sectional characteristics

The Hamada River is bordered by concrete walls on both sides, with the right side having a height of approximately 1 m (Supplementary Figure). Measurements of the river’s cross-sectional area, flow velocity, and discharge were taken on the sampling dates (Supplementary Table). The riverbed on the left side was shallower than on the right, resulting in lower flow velocity on the right side where the water level was higher (Supplementary Figure). Determining a consistent cross section for the entire river, from upstream to downstream, was challenging. The steep riverbank upstream caused significant variations in flow, which limited our ability to measure effectively in those areas.

The sampling frequency was designed to capture the effects of both sunny condition and rainfall events on the river’s water level (Fig. 2). This approach allowed for an assessment of how weather conditions influence the transportation and deposition of plastic waste in the river. During rainy weather, the water level downstream increased approximately sixfold, from about 0.3 m before the rain to 2.0–2.4 m after rainfall, which significantly impacted the hydrodynamic conditions of the river. The changes in the river’s depth and flow velocity made it challenging to collect plastics from the river itself, leading to a shift in sampling to the riverbank.

### Plastic shape, size, and aspect ratio

**Fig. 3:**
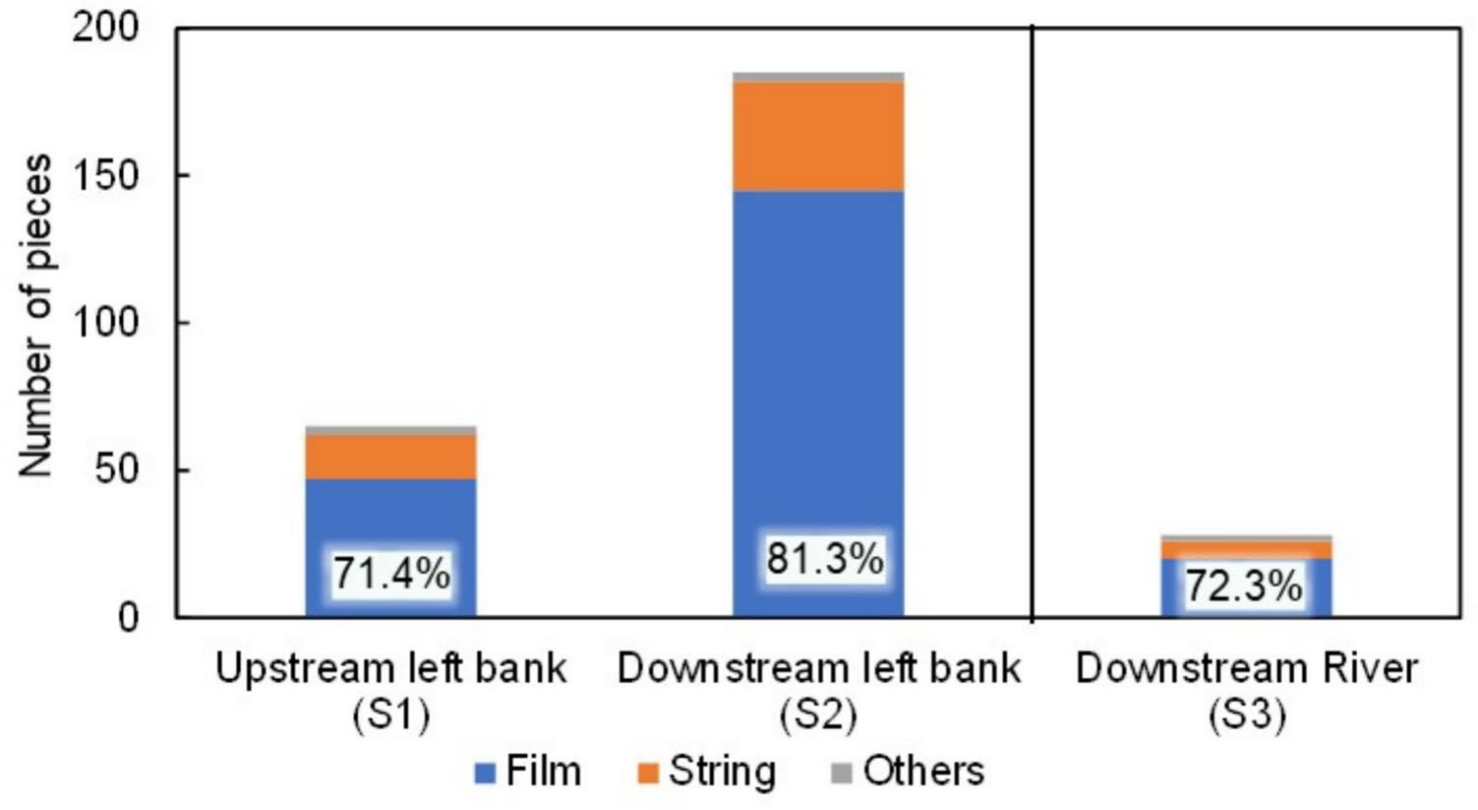
Shapes of macroplastic waste collected from the Hamada River

The shape, size, and aspect ratio of plastics varied, with films making up the majority of plastic waste, representing 70–80% of the total collected from the Hamada River (Fig. 3). The number of macroplastic pieces was higher on the downstream riverbank than on the upstream side (Fig. 3), suggesting that anthropogenic activities may have increased plastic waste emissions from upstream to downstream. As plastic waste in the river was collected during sunny conditions, the number of pieces was lower compared to those on the riverbank, where waste was collected during rainy conditions. However, further surveys are needed to confirm the transportation dynamics of plastic waste between the river and riverbank, especially during rainfall events.

**Fig. 4:**
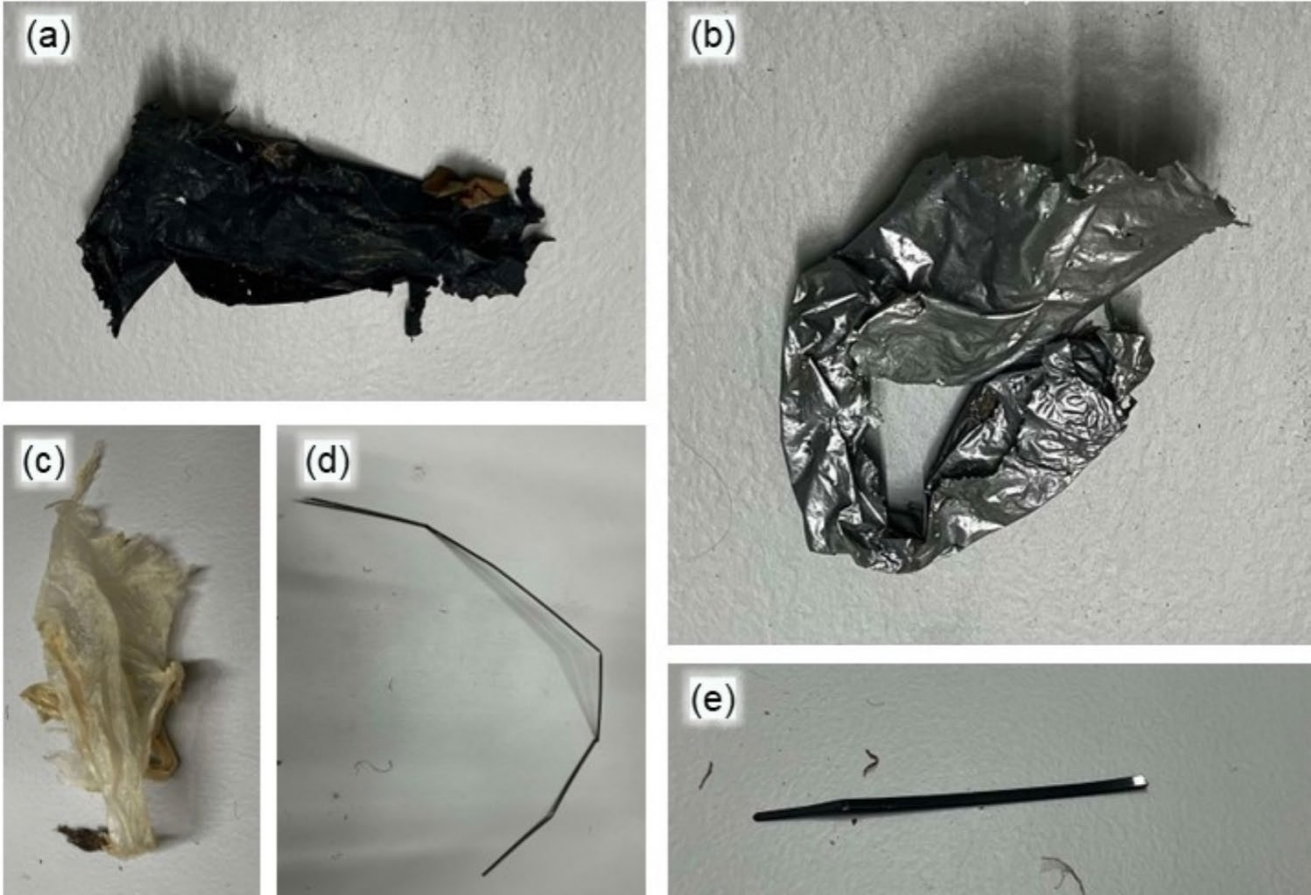
Example images of collected plastic waste. **a** Black mulch film. **b** Grey mulch film. **c** Bag fragment. **d** Rubber band. **e** Hard plastic fragment

Figure 4 shows example images of plastic waste collected in this study. Film waste (Fig. 4a, b), likely originating from mulching or packaging debris, was the dominant macroplastic waste in farmlands in southeast Germany (Piehl et al. 2018). Other plastic waste included fragments (Fig. 4c, d) or hard plastics (Fig. 4e).

**Fig. 5:**
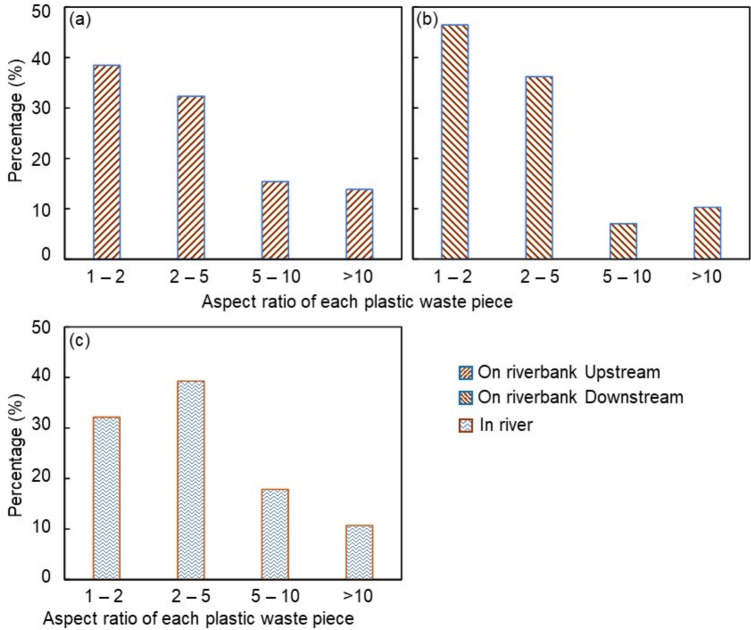
Mass percentage of plastic waste by aspect ratio of each piece. **a** Plastics collected from the upstream riverbank. **b** Plastics collected from the downstream riverbank. **c** Plastics collected in river

On rainy days, plastics found along the riverbank were larger and heavier than those collected from the river during sunny weather, with some downstream samples weighing nearly 60 g. Most plastics collected during rainy weather, 38% from upstream (Fig. 5a) and 46% from downstream (Fig. 5b), had an aspect ratio between 1 and 2, indicating that they were square or rectangular in shape. In contrast, plastics collected from the river on sunny days were smaller, lighter, and more fragmented, with fewer square or rectangular pieces (Fig. 5c). The greatest proportion (39%) of plastics collected from the river had aspect ratios ranging from 2 to 5 (Fig. 5c), suggesting that elongated, stripe-like plastics were prevalent. Higher aspect ratios indicate more elongated plastics, with pieces having ratios greater than 10 possibly originating from rubber bands or threads from degrading bags (Fig. 4c, d). These differences suggest that rain and river flow may cause larger plastic items to break down into smaller, more square or rectangular pieces.

One-way ANOVA with Tukey’s post hoc test revealed that the percentages of samples under different aspect ratios differed significantly between the river and riverbank (*F* = 25.2, *p* = 0.0002 < 0.05). The percentages of plastics were calculated by mass per sample collection site’s area. For plastics floating in the river, the area was the river’s cross-sectional area, whereas for plastics on the riverbank, the area was the quadrat’ s area. The percentages reflect the surface mass distribution density at each of sampling sites.

Although direct comparison between the river and riverbank is limited by different sampling methods, the observed differences in shape and size are likely due to rainfall and high-flow conditions, which facilitate plastic fragmentation (Lee et al. 2013). The higher proportion of elongated items (aspect ratio > 10) found upstream, compared to downstream, may also reflect regional variations in the types of plastics being transported or fragmented.

### Color

**Fig. 6:**
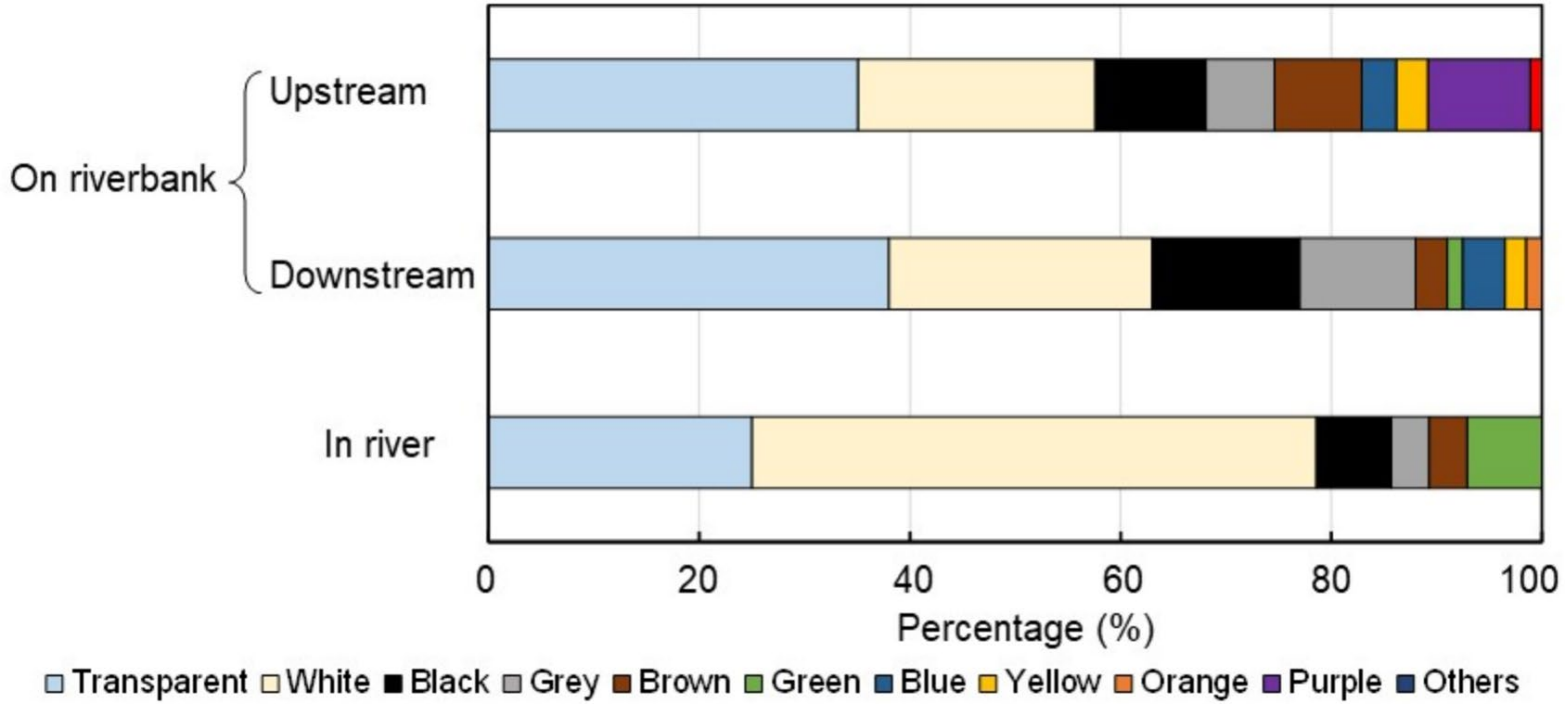
Percentages of macroplastic colors by sampling site

Color variation (Fig. 6) in the plastics collected from the river and along the riverbank was also influenced by weather conditions. More than half (53.6%) of the plastics collected from the river during sunny weather were white, followed by 25% transparent plastics. The remaining plastics were black (7.1%), grey (3.6%), brown (3.6%), or green (7.1%). These colors are commonly associated with anthropogenic sources such as plastic bags and mulch, which are often white. This finding aligns with similar studies conducted on India’s west coast and in Germany’s southeast region (Piehl et al. 2018; Maharana et al. 2020), where white plastics were also predominant.

In contrast, plastics collected from the riverbank during rainy weather showed a broader range of colors. Downstream plastics consisted 38% transparent, 25.1% white, and smaller proportions of black (14%), grey (11%), brown (3%), green (1.6%), blue (4%), yellow (2%), and orange (1.2%). Notably, there was an increase in the percentages of black, grey, and transparent plastics, as well as the amount of mulch waste, on the riverbank during rainy conditions. These three colors are characteristic of mulch, which was found in larger quantities along the riverbank than in the river. At the upstream riverbank, 34.7% of the samples were transparent and 22.7% were white. The remaining plastics included black (10.5%), grey (6.7%), brown (8.3%), blue (3.3%), yellow (3.0%), purple (9.7%), and miscellaneous (1.1%). Interestingly, while the color distribution between upstream and downstream was similar during rainy weather, purple plastics were observed only on the upstream riverbank, suggesting regional differences or specific sources.

Although the sampling methods for the river and riverbank were different, these observations highlight how weather and flow conditions may impact the color diversity of plastics along the riverbank compared to the river. Overall, the percentage of white samples showed the most significant variation between the riverbank and river sites (*F* = 9.09, *p* = 0.0009).

### Macroplastic types and polymer composition

**Fig. 7:**
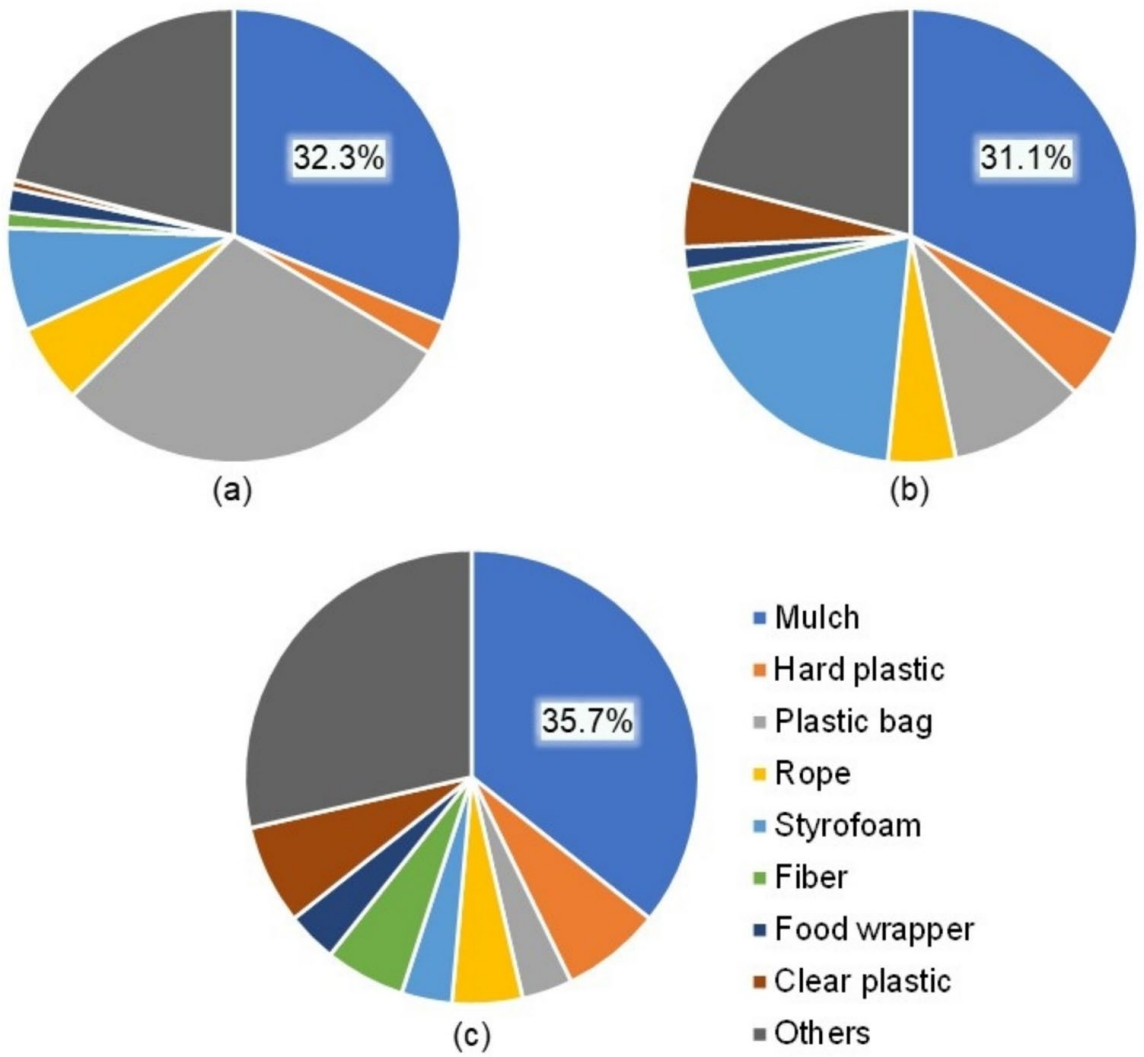
Percentages of item types. **a** Plastics collected from the upstream riverbank. **b** Plastics collected from the downstream riverbank. **c** Plastics collected in the river

The dominant type of plastic debris in the Hamada River and along its riverbank was plastic mulch, with no significant difference between the floating waste in the river and the accumulated waste on the riverbank (*F* = 0.03, *p* = 0.97). However, rain events on riverbank led to an increase in the abundance of plastic bag fragments. In comparison to Hamada River, where most plastics originate from agriculture, the Saigon River in Vietnam is dominated by plastic bags (Lahens et al. 2018), likely due to its proximity to a megacity and a higher prevalence of anthropogenic plastics. The Hamada River produced a minimal amount of food wrappers (1–3.6%), a notable contrast to other macroplastics research where food wrappers are often the most common type of plastic debris (Dalu et al. 2019; Winton et al. 2020). The percentages of macroplastic item types are illustrated in Fig. 7.

**Fig. 8:**
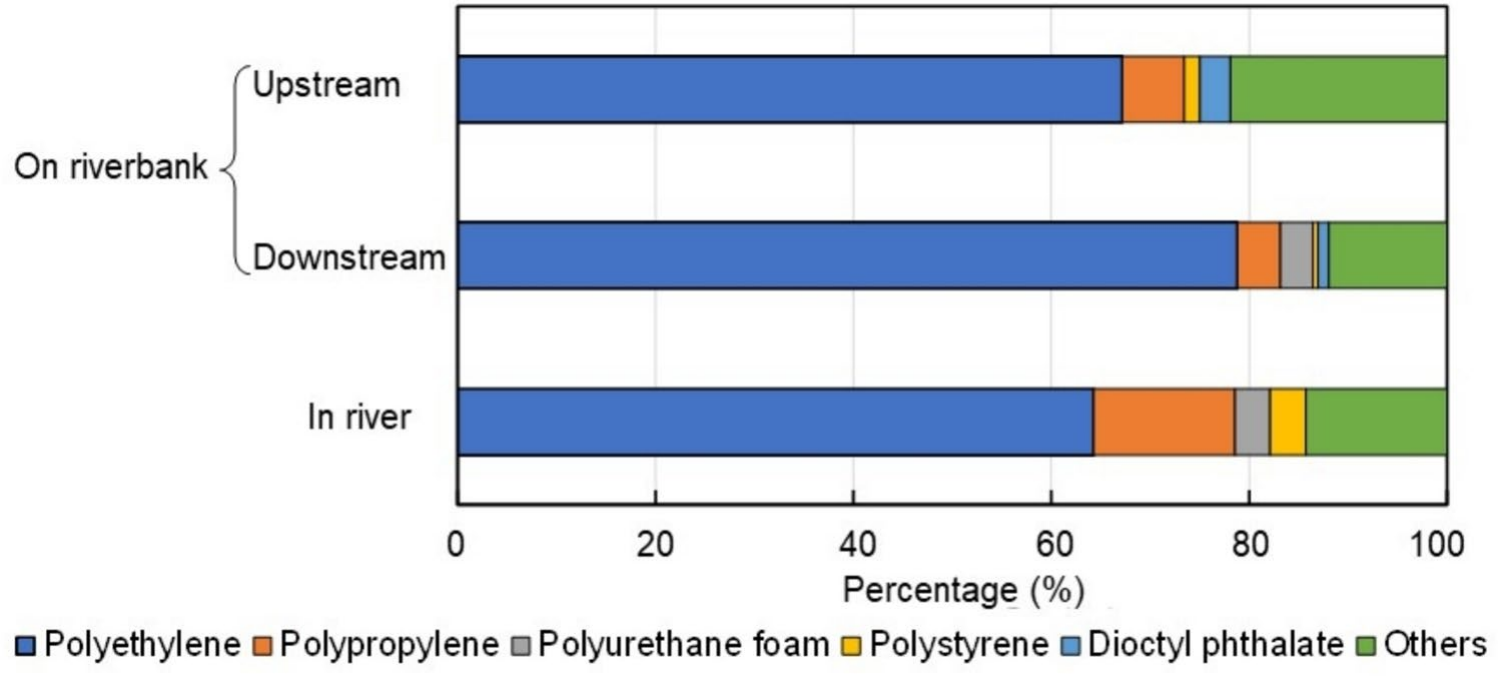
Percentage of polymer compositions in plastic waste by sampling site

During each collection, an average of 92 macroplastic pieces were found, with polyethylene accounting for the majority at an average of 70% (Fig. 8). Polyethylene, mainly in the form of mulch, was by far the dominant polymer type, while polypropylene followed as the second most abundant at 8.3%, commonly found in fertilizer bags, plastic bags, and food wrappers. Polystyrene was also present, primarily from packaging materials. Dioctyl phthalate, an additive used to keep plastics soft, was detected only in samples collected during rainy conditions. Notably, no PET was found in the river system, despite its prevalence in marine environments. Although the differences in sampling locations and methods (river versus riverbank) limit direct comparisons, a *t* test comparing polyethylene and polypropylene revealed significantly different percentages (*t* = 8.735, *p* = 0.012). This suggests that the polymer composition data are consistent with regional trends where agricultural plastics, particularly polyethylene, are prevalent (Piehl et al. 2018; Dalu et al. 2019; Balthazar-Silva et al. 2020).

In Vietnam’s Saigon River, 79% of plastic litter is polyethylene, with polypropylene as the second most abundant polymer at 15% (Balthazar-Silva et al. 2020). This aligns with our results in the Hamada River, where polyethylene also dominated. Furthermore, polyethylene is the major polymer type found in rural agricultural areas of Germany (Piehl et al. 2018).

### Limitations of the study

The study was conducted from the end of May to the end of November, capturing both sunny and rainy days. While this sampling period provided valuable insights, it may have affected the statistical significance, as it did not account for potential variations across different seasons. The plastic waste was collected from a single river, the Hamada River, which is located in close proximity to intensive agricultural activities. This may limit the generalizability of the findings, particularly regarding plastic type and composition, to other rivers or regions with different land-use practices. Additionally, the river is prone to sudden increases in water level during rainfall, and the riverbed is slippery and cobbly, which restricted our team’s ability to enter the river during rain events. As a result, the collection of plastic waste in the river was limited to sunny weather conditions. Furthermore, the accumulated plastic waste on the riverbank was only collected during rainy periods, as the riverbank was almost clear of plastic waste during the sunny days between rain events. The difference on the study’s sampling methods—collecting plastics from the river using a net during sunny conditions and from the riverbank by hand during rainy conditions—presents a limitation in making direct comparisons between the two compartments. The high water levels and flow velocities during rainy conditions made it difficult to sample from the river itself, necessitating a shift to riverbank sampling. Consequently, the data from these two compartments (river and riverbank) should be interpreted separately, with an understanding that different methods were employed under different weather conditions.

While the study focused on macroplastics, it is important to note that microplastics and other smaller plastic particles may also contribute to pollution dynamics, which were not addressed in this study. Future investigations should extend beyond the current sampling period to include more frequent collection of floating plastic waste during rainy conditions and more thorough monitoring of accumulated plastic waste on riverbanks throughout the year. These efforts would help clarify the transportation of plastic emissions from the river to the Pacific Ocean, particularly during high-flow periods. Expanding the geographical scope to include other tributaries of Mikawa Bay and rivers with varying land uses would also provide a broader understanding of plastic waste dynamics in aquatic environments. Additionally, examining the long-term degradation rates of various plastics in the river would help assess the persistence and impact of plastic waste in the ecosystem.

## Conclusions

This study highlights the role of intensive agricultural activities in contributing to macroplastic emissions in the Hamada River, which flows into Mikawa Bay and potentially impacts plastic pollution in the Pacific Ocean. Macroplastics were quantified based on their properties, item types and polymer compositions, with samples collected from both the river and the riverbank during sunny and rainy weather conditions. The findings emphasize the importance of weather-related variations in plastic waste distribution. On sunny days, plastic waste in the river was predominantly lightweight, with film-shaped items being the most common. In contrast, during rainy weather, plastic waste accumulation on the riverbanks increased significantly, with a greater diversity of plastic shapes and colors.

Plastic mulch was the dominant plastic type in both the river and along its riverbank, with polyethylene being the most prevalent polymer, followed by polypropylene. Notably, no PET was found in the Hamada River basin, which contrasts with findings from marine environments where PET is a dominant plastic type. These results suggest that weather conditions, such as rain events, combined with the proximity to agricultural sources, influence the types, sizes, and distribution of plastic waste in rivers. Further research extending to longer-term monitoring, including during rainfall’s high-flow conditions, is needed to fully understand the transport and deposition dynamics of plastic waste in river systems.

## Supplementary Information

Below is the link to the electronic supplementary material.

Supplementary file1 (DOCX 16 KB)

Supplementary file2 (DOCX 124 KB)

## Data Availability

The data supporting the findings of this study are available within the manuscript.
