# Supplementary material for: Quantification of riverine macroplastics in a farmland area in Japan
Source: Environ Sci Pollut Res Int. 2025 Mar 1;32(11):6948–58. doi: 10.1007/s11356-025-36160-6 (PMC11928380; doi:10.1007/s11356-025-36160-6)
Supplement: Supplementary file 1 — Supplementary file1 (DOCX 16 KB) [file 11356_2025_36160_MOESM1_ESM.docx]

**Supplementary Table**. Cross-sectional area, flow velocity, and discharge measurements of the Hamada River on the sampling dates

| Sunny weather sampling date | Cross-sectional area (m^2^) | Flow velocity (m/s) | Discharge (m^3^/s) |
| --- | --- | --- | --- |
| October 19, 2022 | 1.31 | 0.198 | 0.259 |
| October 31, 2022 | 1.41 | 0.181 | 0.254 |
| November 28, 2022 | 1.36 | 0.153 | 0.208 |
