# Supplementary material for: Quantification of riverine macroplastics in a farmland area in Japan
Source: Environ Sci Pollut Res Int. 2025 Mar 1;32(11):6948–58. doi: 10.1007/s11356-025-36160-6 (PMC11928380; doi:10.1007/s11356-025-36160-6)
Supplement: Supplementary file 2 — Supplementary file2 (DOCX 124 KB) [file 11356_2025_36160_MOESM2_ESM.docx]

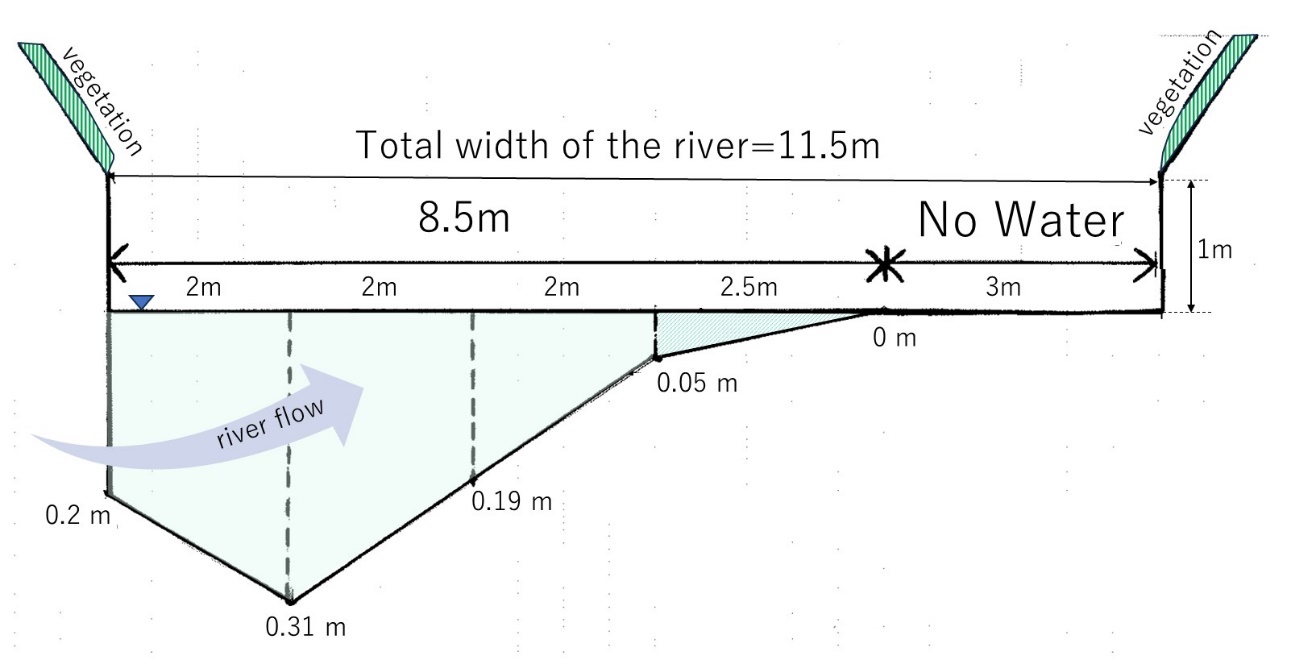


**Supplementary Fig.** Cross-sectional diagram of the Hamada River, as viewed facing the direction of water flow. The riverbed depths are indicated for the date of October 19, 2022.
